# The General Hospitals of the Territorial Army

**Published:** 1908-06-13

**Authors:** 


### THE ROYAL ARMY MEDICAL CORPS SECTION.

### THE GENERAL HOSPITALS OF THE TERRITORIAL ARMY.

IN a previous article we outlined the scheme by which it is proposed to provide for the medical requirements of the new Territorial Force. In the sketch then given of this new medical service, it was pointed out that it would include the formation of field units, of general hospitals, and of a sanitary *personnel*, all of them being on the same lines as those of the Regular Army. Of the field medical units, the first line is to consist of the medical officers of the combatant units with a certain number of regimental stretcher bearers, while the second line will comprise the field ambulances belonging to the various divisions and mounted brigades. In the very full description of a field ambulance, given in a past number of this Journal, it was made quite clear that while this unit receives the sick and wounded from the field troops, it is not intended to serve as a hospital, but is meant only to furnish the immediate care of the sick and the early treatment of the wounded, pending their transference to the general hospitals. It is these latter that constitute the third line of assistance. In them the patients will receive the further necessary treatment, and be placed under conditions similar to those of our best city infirmaries. As it has been decided to make an effort to obtain the services of the medical men of the highest standing in the towns where these hospitals are to be raised to act as physicians and surgeons to them, it is important that the details and arrangements in connection with these general hospitals should be widely known throughout the profession.

The number of these hospitals in connection with the Territorial Army is to be twenty-three, and they are to be formed without delay at various large centres, as set forth in THE HOSPITAL, for May 10 (p. 165). Each hospital will have 520 beds, of which 20 will be for the reception of officers. When opened on mobilisation for the reception of the sick and wounded of the Territorial Army, its staff will consist of the following:—18 physicians and surgeons, an administrator, a quartermaster, a registrar, 91 nurses, and 109 of other *personnel*. In addition to these, it is proposed to attach to each hospital one ophthalmic surgeon, one aural surgeon, and one dental surgeon. Honorary consulting physicians and surgeons may also be appointed.

The position of the civilian physicians and surgeons doing duty with general hospitals has been very clearly laid down. They will be in time of peace *à la suite* members of the Territorial Medical Service, and will possess substantive rank, that of each individual depending on his age and professional seniority. The number of each rank is to be in accordance with war establishments, which will permit of each general hospital having two lieut.-colonels, four majors, and twelve captains. The necessity for such grading of the civilian staff of a general hospital is due to the fact that it has been decided that in time of war the rates of remuneration to be paid to these civilian physicians and surgeons, will be those of the Regular Army, and will be as follows, *per diem*:—

| | In Camp. £ | s. | d. | Not in Camp. £ | s | d. |
|---|---|---|---|---|---|---|
| Lieutenant-colonels ... ... | 2 | 0 | 0 | 1 | 18 | 6 |
| Majors ... ... ... ... | 1 | 12 | 0 | 1 | 11 | 6 |
| Captains ... ... ... ... | 1 | 3 | 0 | 1 | 2 | 6 |
| Lieutenants ... ... ... | 1 | 1 | 0 | 1 | 0 | 3 |

We understand that as yet no definite ruling has been given as to the age limit for physicians and surgeons willing to serve as such on the staffs of these general hospitals. It has been suggested by the War Office that no surgeons over the age of fifty-five and no physicians over the age of sixty should be eligible for these posts, but it is very probable that applicants will come under the same rules as are in existence in the local general hospitals to which they are attached, so that when a physician or surgeon has to retire under the age limit of his hospital, he can no longer serve on the staff of the Territorial General Hospital in that locality.

With the view of rendering it easier for the staffs of local civil hospitals to give their services to these general hospitals, the Director-General has very properly taken special care not to impose conditions of service that would clash with their ordinary professional work. In the first place, by instituting these general hospitals in certain localities, the sick and wounded are brought to the medical men instead of *vice versa*, and in this way the medical staff have not to leave home to carry out their duties. In the next place, it has been fully recognised that the work these physicians and surgeons would voluntarily undertake in the United Kingdom, in time of war, would be exactly similar to that which they carry through every day in civil life. Consequently, it is unnecessary to make any call upon them for annual military training, and the *à la suite* members of the Territorial Medical Service will only be asked to give their assistance in case of invasion or threatened invasion. Further, it has been decided to allow enrolment of physicians and surgeons in excess of the numbers required, thus allowing for reliefs and for the falling out of members who, from any cause, may not be able to come out on mobilisation. All these modifications should render service in the general hospitals of the Territorial Force popular.

The provision of its medical and surgical staff is, however, only a small part of the organising of a large hospital. Its nursing, cooking, clerical, and general duties, have to be provided for by properly trained *personnel*, while the selection of suitable buildings in which, when mobilisation is ordered, the hospital may be installed, and the provision of all necessary equipment for it are matters that demand very careful thought and pre-arrangement. In future articles we hope to consider these points, and to describe the plan by which the Medical Corps of the Territorial Force will provide and train in time of peace, the administrative staffs of these general hospitals, and also a proportion of the more specially trained subordinate *personnel*.

